# Inverse association between glucose-lowering medications and severe hyponatremia: a Swedish population-based case-control study

**DOI:** 10.1007/s12020-019-02160-z

**Published:** 2019-12-25

**Authors:** Henrik Falhammar, Jakob Skov, Jan Calissendorff, Jonatan D. Lindh, Buster Mannheimer

**Affiliations:** 1 Department of Molecular Medicine and Surgery, Karolinska Institutet, Stockholm, Sweden; 2 Department of Endocrinology, Metabolism and Diabetes, Karolinska University Hospital, Stockholm, Sweden; 3 Department of Laboratory Medicine, Division of Clinical Pharmacology, Karolinska University Hospital Huddinge, Karolinska Institutet, Stockholm, Sweden; 4 Department of Clinical Science and Education, Södersjukhuset AB, Karolinska Institutet, Stockholm, Sweden

**Keywords:** Diabetes, Hospitalization, Hyponatremia, SIADH, Adverse reaction

## Abstract

**Context:**

Glucose-lowering medications have occasionally been reported to cause hyponatremia, but the evidence is scarce.

**Objectives:**

To explore the association between glucose-lowering medications and severe hyponatremia.

**Design, setting, and participants:**

Subjects hospitalized with a principal diagnosis of hyponatremia (*n* = 14,359) were compared with matched controls (*n* = 57,383). Data were derived by linkage of national population-based registers. Multivariable logistic regression adjusting for co-medication, diseases, previous hospitalizations, and socioeconomic factors was used to explore the association between hospitalization for hyponatremia and the use of different glucose-lowering medications. Furthermore, newly initiated (≤90 days) and ongoing use was investigated separately.

**Main outcome measures:**

Hospitalization due to hyponatremia.

**Results:**

The unadjusted ORs (95% CI) for hospitalization due to hyponatremia were 1.41 (1.29–1.54) for insulins, 1.38 (1.27–1.50) for metformin, and 1.22 (1.07–1.38) for sulfonylureas. However, after adjustment for confounding factors the association was consistently reversed. Thus, for any glucose-lowering medication the adjusted OR was 0.63 (0.58–0.68). For insulins, metformin and sulfonylureas, adjusted ORs (95% CI) were 0.58 (0.52–0.65), 0.81 (0.72–0.90) and 0.81 (0.69–0.94), respectively. Odds ratios for newly initated medications were overall higher while those for ongoing treatment were further decreased. Thus, adjusted ORs (95% CI) for ongoing treatment with insulins, metformin, and sulfonylureas were 0.54 (0.48–0.61), 0.82 (0.73–0.91) and 0.78 (0.66–0.92).

**Conclusions:**

Glucose-lowering medications did not increase the risk for hospitalization due to severe hyponatremia. In fact, the association was inverse across all investigated drugs. The association may be mediated by pharmacologic mechanisms, but the uniform effects across drug-classes suggest properties of the diabetic disease are of importance.

## Introduction

Electrolyte disturbances are common in patients, with up to 30% of all hospitalized patients displaying hyponatremia [1, 2]. Symptoms of hyponatremia range from mild, non-specific such as lethargy, agitation, and confusion to severe life-threatening such as seizures, coma and death due to brain edema [3–7]. Drugs such as thiazide diuretics, antidepressants, antiepileptic drugs, and antipsychotics are common causes of hyponatremia [8–16].

Electrolyte disturbances are frequent in diabetes mellitus, mainly due to diuretics, acid-base disturbances, and poor glycemic control. Translocational hyponatremia is common in hyperglycemia. The osmotic effect of excess glucose in the extracellular compartment causes a shift of water from the intracellular to the extracellular space, diluting the plasma sodium [6, 17, 18]. This form of hyponatremia is hypertonic and does not reflect a primary dysregulation of water and sodium balance, but rather a dysregulation of glucose. However, hypotonic hyponatremia, reflecting a primary dysregulation in water balance, has been described in occasional case reports and smaller observational studies for sulfonylureas [19–25] as well as insulin [26], metformin [27], and thiazolidinediones [28]. Cell studies also indicate that insulin has a direct effect on water homeostasis [29]. In contrast, based on an artificial syndrome of inappropriate ADH-secretion (SIADH) model, the SGLT2-inibitor empagliflozin, with its increased urine excretion through osmotic diuresis, has been suggested as a new treatment option for SIADH-induced hyponatremia [30]. Thus, the evidence about glucose-lowering medications and hyponatremia remains insufficient.

The primary aim of this study was to investigate the association between treatment with glucose-lowering medications and hospitalization due to severe hyponatremia. Secondarily, we aimed to evaluate newly initiated versus ongoing use of glucose-lowering medications to investigate any time-dependent effects.

## Methods

The study was a retrospective case-control study of the Swedish general population. The principal diagnosis of each admission was used since this reflects the main cause of the hospitalization. The *International Classification of Diseases*, 10th Revision (ICD10), is used by all physicians in Sweden to code all hospitalizations and outpatient visits [31]. Adult patients (≥18 years) hospitalized with a first-ever (defined as not occurring since 1 January 1997) principal ICD10 code of E87.1 (hyponatremia) or E22.2 (SIADH secretion) in The National Patient Register (NPR) (see below) were classified as cases during the study period (1 October 2005 to 31 December 2014). Four age-, sex- and municipality-matched controls per case with no previous diagnosis of hyponatremia (since 1 January 1997) were randomly identified from the Total Population Register. The principal diagnosis of hyponatremia, with sodium levels corrected for glucose levels, was validated in one of the larger hospitals. More details on this process can be found elsewhere [11].

All variables in the multiple logistic regression analysis are presented in Table 1. Potential confounders for hyponatremia were identified using ICD10 codes, Anatomical Therapeutic Chemical codes, and parameters from the Longitudinal integration database for health insurance and labor market studies (LISA)-register [11]. Exposure to glucose-lowering medications was defined as a documented dispensation within 90 days prior to the index date, i.e., the date of admission due to hyponatremia. In the matched controls, the index date was the admission date of their case. Almost all drugs used for long-term treatment are dispensed every 90 days in Sweden [11]. Adjustment for concurrent disorders was done since 1 January 1997 to the index date, with the exception of infectious diseases (which were adjusted for within 90 days before the index date). Newly initiated use of glucose-lowering medications was defined as treatment introduced within 90 days before the index date and at least 12 months of no exposure beforehand. The definition of ongoing use of glucose-lowering medications also required one or more dispensations in the period 91–454 days before the index date.

**Table 1:** Variables included in the multiple logistic regression analysis and their definition

Variables	Codes
	ATC codes beginning with
Drugs of primary interest	
Insulins	A10A
Metformin	A10BA02
Sulfonylureas	A10BB
DPP4-inhibitors	A10BH
GLP1-analogs	A10BX04, A10BX07, A10BX10, A10BX13, A10BX14, A10BJ
SGLT-2 inhibitors	A10BX09, A10BX11, A10BX12, A10BK
Thiazolidinediones	A10BG
Meglitinides	A10BX02, A10BX03
Antiepileptic drugs	
Carbamazepine	N03AF01
Oxcarbazepine	N03AF02
Phenytoin	N03AB02
Valproate	N03AG01
Lamotrigine	N03AX09
Levetiracetam	N03AX14
Gabapentin	N03AX12
Diuretics and drugs on the renin-angiotensin system	
Furosemide	C03C
Thiazides	C03A, C09BA, C09DA, C03EA
Agents acting on the renin-angiotensin system	C09
Antibiotics	
Fluoroquinolones	J01MA
Macrolides	J01FA
Trimethoprim sulfamethoxazole	J01EE
Antidepressants	
SSRIs	N06AB
Tricyclic antidepressants	N06AA
Other antidepressants	N06AX
Other drugs	
Amiodarone	C01BD01
Desmopressin	H01BA02
Proton pump inhibitors	A02BC, A02BD06
Antipsychotics (including lithium)	N05A
	ICD10 codes beginning with
Renal diseases	
Renal insufficiency	N17-19, procedure codes DR016, DR024, KAS00, KAS10, KAS20
Infections	
Sepsis	A41
Pneumonia	J18
Meningitis	G00–G07
Heart and vascular diseases	
Ischemic heart disease	I20–25
Congestive heart failure	I50
Cerebrovascular diseases	I60–64, I69
Gastrointestinal diseases	
Pancreatic disease	K85, K860-1
Inflammatory bowel disease	K50-51
Liver diseases	K70-77 Procedure codes JJB, JJC
Other diseases	
Hypothyroidism	E03, E06.3
Malnutrition	E43.9, E41.9
COPD	J44
Pulmonary embolism	I26
Malignancy	C
	Combination of ATC- and ICD10 codes, each beginning with
Alcoholism	ATC: N07BB ICD10: E244, F10, G312, G621, G721, I426, K292, K70, K860, O354, P043, Q860, T51, Y90-91, Z502, Z714
Adrenal insufficiency	ATC: H02AA, H01BA ICD10: E27.1, E27.2, E27.3, E27.4, E25
Socioeconomic factors	
Education	Increasing levels of education from 1–6, continuous variable
Income	Income in Swedish crowns during 1 year per family, continuous variable
Unemployment	Number of days, continuous variable
Proxy for frailty	
Drug use	Number of dispensed drugs 90 days prior to index date, categorized into <4, 4–7, 8–12 and >12 drugs
Duration of hospitalization	≥3 days

A diagnosis of diabetes (ICD10 codes E10-14) was not included in the multiple regression analysis

*SSRIs* selective serotonin reuptake inhibitors, *COPD* chronic obstructive pulmonary disease

Using the unique Swedish personal identification number linkage between the population-based registers was possible. The NPR, The Swedish Prescribed Drug Register (SPDR) and the LISA-register were all utilized in the linkage [31–33]. The NPR contains all the ICD10 codes (since 1997), while the SPDR contains all prescriptions dispensed in Sweden (since 1 July 2005). The LISA-register was used to control for socioeconomic variables. The Regional Ethical Review Board in Stockholm approved the study and as this was a retrospective epidemiological study, formal consent was waived.

### Statistical analysis

The associations between hospitalization due to hyponatremia and glucose-lowering medications were analyzed by means of univariable and multivariable logistic regression. In these models, the reference group was defined as individuals unexposed to any of the drugs or variables adjusted for (see Table 1). The associations between glucose-lowering medications and hospitalization due to hyponatremia in cases and controls were reported as unadjusted and adjusted odds ratios (OR), with 95% confidence intervals (95% CI). *P* values < 0.05 were considered statistically significant. For all analyses R version 3.3.2 was used [34].

## Results

In 14,359 adult individuals the principal discharge diagnosis had been hyponatremia and they were matched to 57,382 controls identified in the Total Population Register. Overall, 72% were females and the median age in the cohort was 76 years (range 18–103). In Table 2, a selection of medical conditions and the use of glucose-lowering medications at baseline (index date) are presented in the entire group as well as in individuals below or over 65 years of age. The most frequent medical disorders besides hyponatremia were malignancy, ischemic heart disease, alcoholism, and diabetes. Among individuals <65 years old, females and chronic disease were less frequent while alcoholism was more prevalent compared with individuals ≥65 years old. In total, 10.1% of the cases had been recently dispensed a glucose-lowering medication compared with 7.5% of the controls. The most common glucose-lowering medications in both cases and controls were metformin (*n* = 3027), insulin (*n* = 2510), and sulfonylureas (*n* = 1359), while the number on meglitinides (*n* = 221), DPP4-inhibitors (*n* = 138), thiazolidinediones (*n* = 78), or GLP-1 analogs (*n* = 78) was lower. In the two individuals using a SGLT-2 inhibitor no OR was calculated.

**Table 2:** Medical characteristics (selection of items from Table 1) in addition to glucose-lowering medication use among cases (hospitalized with a principal diagnosis of hyponatremia) and controls at index date

	Number of total cases (*n* = 14,359)	Number of total controls (*n* = 57,382)	Number of cases <65 years (*n* = 3421)	Number of controls <65 years (*n* = 13,684)	Number of cases ≥65 years (*n* = 10,938)	Number of controls ≥65 years (*n* = 43,698)
Median age (range)	76 years (18–103)	76 years (18–103)	57 years (18–64)	57 years (18–64)	81 years (65–103)	81 years (65–103)
Females	10,336 (72.0%)	41,299 (72.0%)	1822 (53.3%)	7288 (53.3%)	8514 (77.8%)	34,011 (77.8%)
Diagnosis						
Malignancy	3826 (26.6%)	11251 (19.6%)	595 (17.4%)	1144 (8.4%)	3231 (29.5%)	10,107 (23.1%)
Ischemic heart disease	2808 (19.6%)	7880 (13.7%)	272 (8.0%)	469 (3.4%)	2536 (23.2%)	7411 (17.0%)
Alcoholism	2285 (15.9%)	1028 (1.8%)	1301 (38.0%)	462 (3.4%)	984 (9.0%)	566 (1.3%)
Diabetes	2273 (15.8%)	4995 (8.7%)	552 (16.1%)	493 (3.6%)	1721 (15.7%)	4502 (10.3%)
Congestive heart failure	1900 (13.2%)	4493 (7.8%)	226 (6.7%)	105 (0.8%)	1674 (15.3%)	4388 (10.0%)
Cerebrovascular diseases	1884 (13.1%)	4540 (7.9%)	319 (9.3%)	202 (1.5%)	1565 (14.3%)	4338 (9.9%)
COPD	1477 (10.3%)	1958 (3.4%)	345 (10.1%)	132 (1.0%)	1132 (10.3%)	1826 (4.2%)
Hypothyroidism	1439 (10.0%)	2396 (4.2%)	205 (6.0%)	166 (1.2%)	1234 (11.3%)	2230 (5.1%)
Adrenal insufficiency	586 (4.1%)	340 (0.6%)	168 (4.9%)	37 (0.3%)	424 (3.9%)	303 (0.7%)
Renal diseases	631 (4.4%)	1098 (1.9%)	185 (5.4%)	73 (0.5%)	446 (4.1%)	1025 (2.3%)
Liver diseases	553 (3.9%)	417 (0.7%)	254 (7.4%)	113 (0.8%)	399 (3.6%)	304 (0.7%)
Pancreatic disease	327 (2.3%)	513 (0.9%)	136 (4.0%)	74 (0.5%)	191 (1.7%)	439 (1.0%)
Inflammatory bowel disease	285 (2.0%)	533 (0.1%)	113 (3.3%)	142 (1.0%)	172 (1.6%)	391 (0.9%)
Glucose-lowering medication, total						
Insulins	648 (4.5%)	1862 (3.2%)	215 (6.3%)	209 (1.5%)	433 (4.0%)	1653 (3.8%)
Metformin	768 (5.3%)	2259 (3.9%)	185 (5.4%)	357 (2.6%)	583 (5.3%)	1902 (4.4%)
Sulfonylureas	316 (2.2%)	1043 (1.8%)	44 (1.3%)	86 (0.6%)	272 (2.5%)	957 (2.2%)
DPP4-inhibitors	31 (0.2%)	107 (0.2%)	4 (0.1%)	22 (0.2%)	27 (0.2%)	85 (0.2%)
GLP1-analogs	9 (0.06%)	34 (0.06%)	2 (0.06%)	11 (0.08%)	7 (0.06%)	23 (0.05%)
SGLT-2 inhibitors	1 (0.007%)	1 (0.002%)	0 (0%)	1 (0.007%)	1 (0.09%)	0 (0%)
Thiazolidinediones	21 (0.1%)	57 (0.1%)	6 (0.2%)	14 (0.1%)	15 (0.1%)	43 (0.1%)
Meglitinides	55 (0.4%)	166 (0.3%)	6 (0.2%)	14 (0.1%)	42 (0.4%)	145 (0.3%)
Any glucose-lowering medication	1446 (10.1%)	4320 (7.5%)	378 (11.0%)	551 (4.0%)	1068 (9.8%)	3769 (8.6%)
Glucose-lowering medication, newly initiated treatment						
Insulins	78 (0.5%)	107 (0.2%)	24 (0.7%)	9 (0.07%)	54 (0.5%)	98 (0.2%)
Metformin	48 (0.3%)	150 (0.3%)	11 (0.3%)	28 (0.2%)	37 (0.3%)	122 (0.3%)
Sulfonylureas	34 (0.2%)	91 (0.2%)	7 (0.2%)	10 (0.07%)	27 (0.2%)	81 (0.2%)
DPP4-inhibitors	7 (0.05%)	20 (0.03%)	1 (0.03%)	6 (0.04%)	6 (0.05%)	14 (0.03%)
GLP1-analogs	4 (0.03%)	7 (0.01%)	1 (0.03%)	4 (0.03%)	3 (0.03%)	3 (0.007%)
SGLT\ inhibitors	1 (0.007%)	0 (0%)	0 (0%)	0 (0%)	1 (0.009%)	0 (0%)
Thiazolidinediones	2 (0.01%)	3 (0.005%)	0 (0%)	1 (0.007%)	2 (0.02%)	2 (0.005%)
Meglitinides	8 (0.06%)	16 (0.03%)	1 (0.03%)	2 (0.01%)	7 (0.06%)	14 (0.03%)
Any glucose-lowering medication	91 (0.6%)	206 (0.4%)	28 (0.8%)	29 (0.2%)	63 (0.6%)	177 (0.4%)

*COPD* chronic obstructive pulmonary disease

**Fig. 1:**
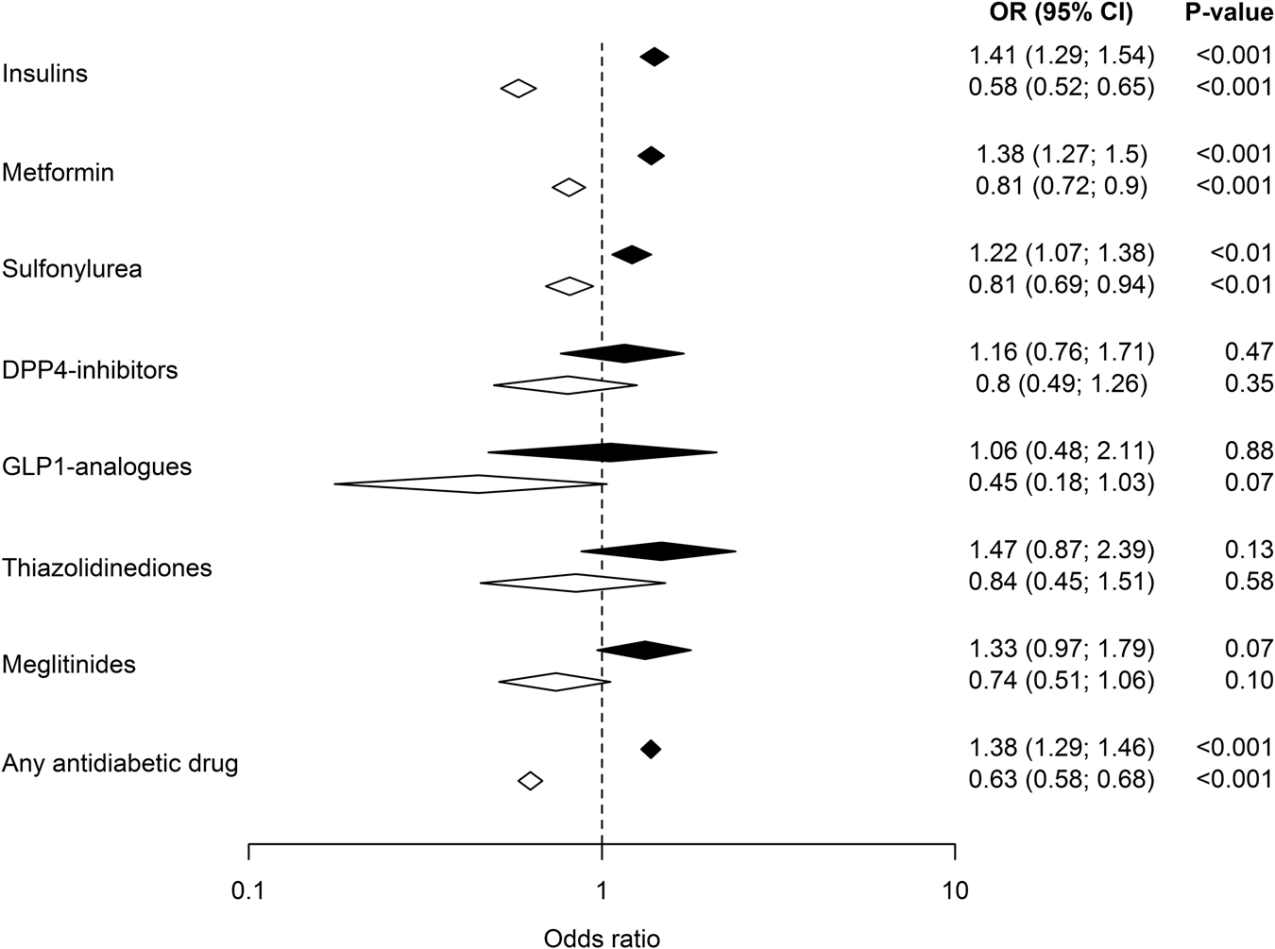
The crude (white) and adjusted (black, all variables in Table 1) odds ratio (OR), including 95% confidence intervals (95% CI) for hospitalization due to severe hyponatremia in patients on different glucose-lowering medication (newly/ongoing)

The association between exposure to glucose-lowering medications and hyponatremia hospitalization is presented in Fig. 1. Compared with controls, the unadjusted OR for hospitalization due to hyponatremia for any glucose-lowering medication compared with controls was 1.38 (1.29–1.46). The unadjusted ORs for (95% CI) was 1.41 (1.29–1.54) for insulins, 1.38 (1.27–1.50) for metformin, and 1.22 (1.07–1.38) for sulfonylureas. However, after adjustment for confounding factors the association was consistently reversed. Thus, for any glucose-lowering medication the adjusted OR was 0.63 (0.58–0.68). ORs (95% CI) for insulins, metformin, and sulfonylureas were 0.58 (0.52–0.65), 0.81 (0.72–0.90), and 0.81 (0.69–0.94), respectively.

**Fig. 2:**
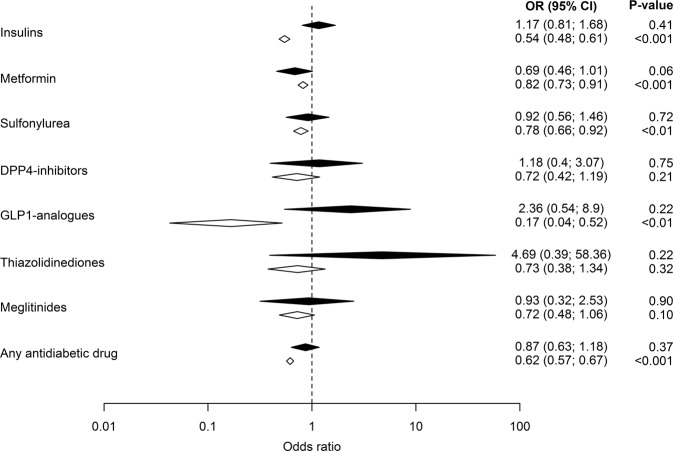
The odds ratio (OR), including 95% confidence intervals (95% CI) for hospitalization due to severe hyponatremia in patients with ongoing (white) and newly initiated glucose-lowering medication (black). All ORs have been adjusted for the confounding factors in Table 1

In Fig. 2 the effect of newly initiated glucose-lowering medication use versus ongoing therapy (adjusted ORs) is presented. OR for newly initiated medications were overall higher while ongoing treatment was further decreased. Thus, adjusted ORs (95% CI) for ongoing treatment with insulins, metformin, and sulfonylureas were 0.54 (0.48–0.61), 0.82 (0.73–0.91), and 0.78 (0.66–0.92).

## Discussion

This is the first population-based case-control study reporting on glucose-lowering medications and hospitalization due to severe hyponatremia. Glucose-lowering medications were associated with severe hyponatremia requiring hospitalization. However, after adjusting for confounding factors the associations were consistently reversed with OR ranging from 0.45 to 0.81. These inverse associations were more pronounced for ongoing compared with newly initiated treatment.

Glucose-lowering medication-induced hyponatremia has previously been reported in occasional case reports or, for sulfonylurea, also in smaller observational studies [19–28]. In one study, published in 1983, 6.3% of 176 patients treated with chlorpropamide developed hyponatremia during a mean follow-up period of 7.4 years, compared with 0.6% in 162 patients treated with tolbutamide or glibenclamide [20]. However, adjustment for factors known to induce hyponatremia had not been done. The results of the present study are in line with these studies showing a crude increased association between glucose-lowering medications and hospitalization due to severe hyponatremia. However, after adjustment for potential confounding factors, the effect not only disappeared but consistently was reversed suggesting a protective effect.

The results indicated a temporal association between initiation of glucose-lowering medication and hospitalization due to hyponatremia, i.e., the risk of severe hyponatremia was higher for drugs newly initiated versus ongoing treatment, especially for insulin and GLP-1 analog use. This may be explained by the fact that a subclinical diabetes is often revealed by severe diseases such as pneumonia, pyelonephritis, or acute pancreatitis [35], which in turn may increase the risk for a near future hospitalization due to hyponatremia.

Theoretically, possible mechanisms may be associated to the pharmacologic action of respective drug, or to mechanisms attributed to the underlying diabetes. Glibenclamide has been reported to induce diuresis [23], probably due to antagonistic effect to ADH [36]. It has even been used to treat SIADH [37]. Insulins can also cause fluid retention but have antinatriuretic properties as well and increase sodium reabsorption by effects on renal sodium channels [38]. The mechanism of action for metformin is not entirely clear, and a putative protective effect against severe hyponatremia may be more difficult to explain. Although the use of the remaining glucose-lowering medications was lower and the estimations therefore more uncertain resulting in wider confidence intervals, the overall tendency went in the same direction towards an inverse association with hospitalization for severe hyponatremia.

The common denominator among the drugs investigated in the present study is that they are used to treat individuals with diabetes. Their chemical structures and mechanism of action are very different. The fact that the association was inverse across all drugs despite their heterogeneity, may suggest that factors other than the glucose-lowering medication per se could be considered. The most likely explanation is perhaps the diabetic disease. In other words, diabetes itself may protect against severe hyponatremia requiring hospitalization. The most plausible mechanism may involve reversing water retention caused by SIADH, the most common cause of hyponatremia [6]. Hyperglycemia with plasma glucose levels above the renal threshold for reabsorption result in osmotic diuresis and reduced extracellular volume. Thus, this mechanism potentially counteracts the effect of SIADH resulting in a decreased risk for severe hyponatremia.

This study has some further strengths and limitations. The major strength is the population-based design with the inclusion of all patients admitted with a principal diagnosis of hyponatremia, i.e., severe hyponatremia, in the entire country during almost a decade. The major limitation on the other hand was that no plasma sodium levels were available. However, only clinically relevant hyponatremia was included since we used the Swedish physicians’ mandatory selection of principal diagnosis. We believe this is an advantage compared with studies including individuals with hyponatremia as a secondary diagnosis, diagnoses made in the secondary care [9], or patients with a mild to moderate hyponatremia regardless of symptoms [10]. In a previous validation of 104 patients hospitalized with a principal diagnosis of hyponatremia, we found that 89% of cases had a correct principal diagnosis, with a mean plasma sodium level of 121 mmol/L [11]. What is more, 77% had been exposed to levels below <125 mmol/L [11], i.e., the level defining profound hyponatremia [6], further showing the clinical relevance of the outcome used. To investigate the possibility that the hyponatremia in a proportion of cases was explained by translocational hyponatremia due to a badly controlled diabetes we reviewed the 104 hospitalizations in the validation cohort. It turned out that 15 patients had been diagnosed with diabetes at the point of hospitalization. Mean (range) HbA1c was 66 (43–128) mmol/L. Crude and glucose corrected (range) sodium levels were 118 (110–128) and 120 (110–130) mmol/L, respectively. Furthermore, in none of the 15 cases the hyponatremia was caused by translocational hyponatremia.

However, among the vast majority of patients outside the validation cohort we did not have access to blood glucose levels. Therefore, we cannot exclude that patients suffering from translocational hyponatremia due to hyperglycemia erroneously received a principal diagnosis of hyponatremia. While theoretically possible, this is an unlikely explanation to our findings; Firstly, for translocational hyponatremia to cause a serum sodium levels to drop to 125 mmol/L or lower, glucose levels would have to be in excess of 40 mmol/L, corresponding to a glycemic crisis. In this setting a principal diagnosis of hyponatremia seems unlikely. Secondly, a misdiagnosis of translocational hyponatremia for hypotonic hyponatremia would have resulted in a positive bias that would have caused us to overestimate rather than underestimate the negative associations observed in this study. Finally, although we corrected for various of concomitant conditions and medications, the risk for residual confounding cannot be excluded.

There are several important clinical implications of the present study. Firstly, severe hyponatremia may occur despite rather than due to any glucose-lowering medication or underlying diabetic disease. Secondly, the current study exemplifies the importance of postmarketing surveillance to reveal hitherto unrecognized properties of medications and to evaluate the real-world effectiveness and safety of the drug [39, 40].

In conclusion, glucose-lowering medications did not increase the risk for hospitalization due to severe hyponatremia. In fact, the associations were consistently inverse across all investigated drugs. Theoretically, this association may be mediated by pharmacologic mechanisms, but the uniform effects across all drug-classes suggests properties of the diabetic disease are of importance.
